# Grid cells in rats deprived of geometric experience during development

**DOI:** 10.1073/pnas.2310820120

**Published:** 2023-10-02

**Authors:** Ingvild Ulsaker-Janke, Torgeir Waaga, Tanja Waaga, Edvard I. Moser, May-Britt Moser

**Affiliations:** ^a^Kavli Institute for Systems Neuroscience and Centre for Algorithms in the Cortex, Norwegian University of Science and Technology, 7491 Trondheim, Norway

**Keywords:** hippocampus, entorhinal cortex, spatial coding, development

## Abstract

The medial entorhinal cortex (MEC) is part of the brain’s network for dynamic representation of location. The most abundant class of neurons in this circuit is the grid cell, characterized by its periodic, hexagonally patterned firing fields. While in developing animals some MEC cell types express adult-like firing patterns already on the first exposure to an open spatial environment, only days after eye opening, grid cells mature more slowly, over a 1-to-2-wk period after the animals leave their nest. Whether the later emergence of a periodic grid pattern reflects a need for experience with spatial environments has not been determined. We here show that grid-like firing patterns continue to appear during exploration of open square environments in rats that are raised for the first months of their life in opaque spherical environments, in the absence of stable reference boundaries to guide spatial orientation. While strictly periodic firing fields were initially absent in these animals, clear grid patterns developed after only a few trials of training. In rats that were tested in the same open environment but raised for the first months of life in opaque cubes, with sharp vertical boundaries, grid-like firing was from the beginning indistinguishable from that of nondeprived control animals growing up in large enriched cages. Thus, although a minimum of experience with peripheral geometric boundaries is required for expression of regular grid patterns in a new environment, the effect of restricted spatial experience is overcome with short training, consistent with a preconfigured experience-independent basis for the grid pattern.

## Significance

An animal’s current position is encoded by specialized neurons in the medial entorhinal cortex, including grid cells and head direction cells. The location and direction-coding properties of these cells can be observed at early postnatal age when animals explore an environment for the first time. While this observation points to strong maturational components, the contribution of spatial experience has not been determined. In this study, we show that minimizing geometric experience throughout postnatal development retards but does not prevent the formation of grid cells at adult age. Rats raised for the first few months in opaque spherical environments do have grid cells, although stable periodic firing may require a few days of experience.

The purpose of the present study was to assess the impact of early geometric experience on the emergence of grid cells (1, 2) and head direction cells (3–5) in the position-coding circuits in the medial entorhinal cortex (MEC). The work was inspired by decades of investigations in the visual cortex, dating back to the early 1960s, in which it was established that the functional correlates of neurons in this area are sensitive to the animal’s experience during early postnatal life (6). In young animals, interventions such as monocular deprivation were shown to shift the responsiveness of normally binocularly driven V1 neurons to the open eye, resulting in a substantial loss of binocularly excited neurons as well as ocular dominance columns (7, 8). This effect was observed also with less severe and more specific interventions, such as when the two eyes were occluded alternatingly or when one eye was rotated to minimize overlap between the two visual fields (9, 10). In the same way, although to a lesser extent, orientation tuning of visual cortex cells was shown to be influenced by the orientation of visual inputs that animals received during early experience (11–13), and self-actuated movement was shown to be necessary for the development of visually guided behavior (14). While from the beginning, there was little doubt that visual cortex neurons had significant binocular and orientation-tuning properties already prior to experience, in very young animals (15), the subsequent decades of experience-manipulation studies established a framework for understanding how cortical circuit development is shaped by correlated and patterned activity during postnatal experience. We have here used this framework as a starting point for asking whether certain kinds of experience are similarly required for the functional maturation of a high-level nonsensory cortex, the MEC, a brain system where neurons express strong correlates between neuronal activity and features of the external spatial environment (1–5, 16, 17).

Converging evidence suggests that while entorhinal principal cells are born prenatally and major functional connections are present already at P6 to P10 (18–20), the system continues to mature during the first weeks of postnatal life (20–22). The development of the network is accompanied by maturation of the space-tuning properties of neurons in the circuit. When rat pups take their first exploratory steps outside the nest around the time of eye opening, 2 wk after birth, head direction cells and border cells already have firing characteristics that are indistinguishable from those of adult animals (23–25). In contrast, grid cells appear to follow a slower developmental trajectory. Although many MEC cells exhibit spatial periodicity above the chance level during the animal’s first exploration experiences, the number of cells with stable rotationally symmetric firing fields does not reach adult levels until several days later (26–28). This final maturation stage of the grid cells coincides with the time when rat pups become skilled navigators (29–31), raising the possibility that the expression of grid cell patterns depends not only on the assembly of prespecified connectivity within the microcircuit (20–22), but also on navigational learning and experience.

A defining feature of all environments is the configuration of local boundaries. In the present study, inspired by the classical deprivation studies in the visual cortex (6–15), we thus set out to test the prediction that young rats require experience with salient local boundaries to express stable, symmetric grid patterns. Rats were raised for more than 2 mo in family groups in three geometrically different environments: i) an empty opaque sphere, ii) an empty opaque cube, or iii) a spatially complex transparent cage full of objects. The spherical environment minimized the presence of stable vertical boundaries that could be used for orientation of spatial maps. The cube and the enriched cage, in contrast, had clearly defined boundaries. At adult age, rats from the three housing conditions were tested for the first time in an external environment, a square open field, in order to determine whether hexagonally periodic firing fields develop in grid cells regardless of the animal’s exposure to environmental geometry during development.

## Results

**Fig. 1.**
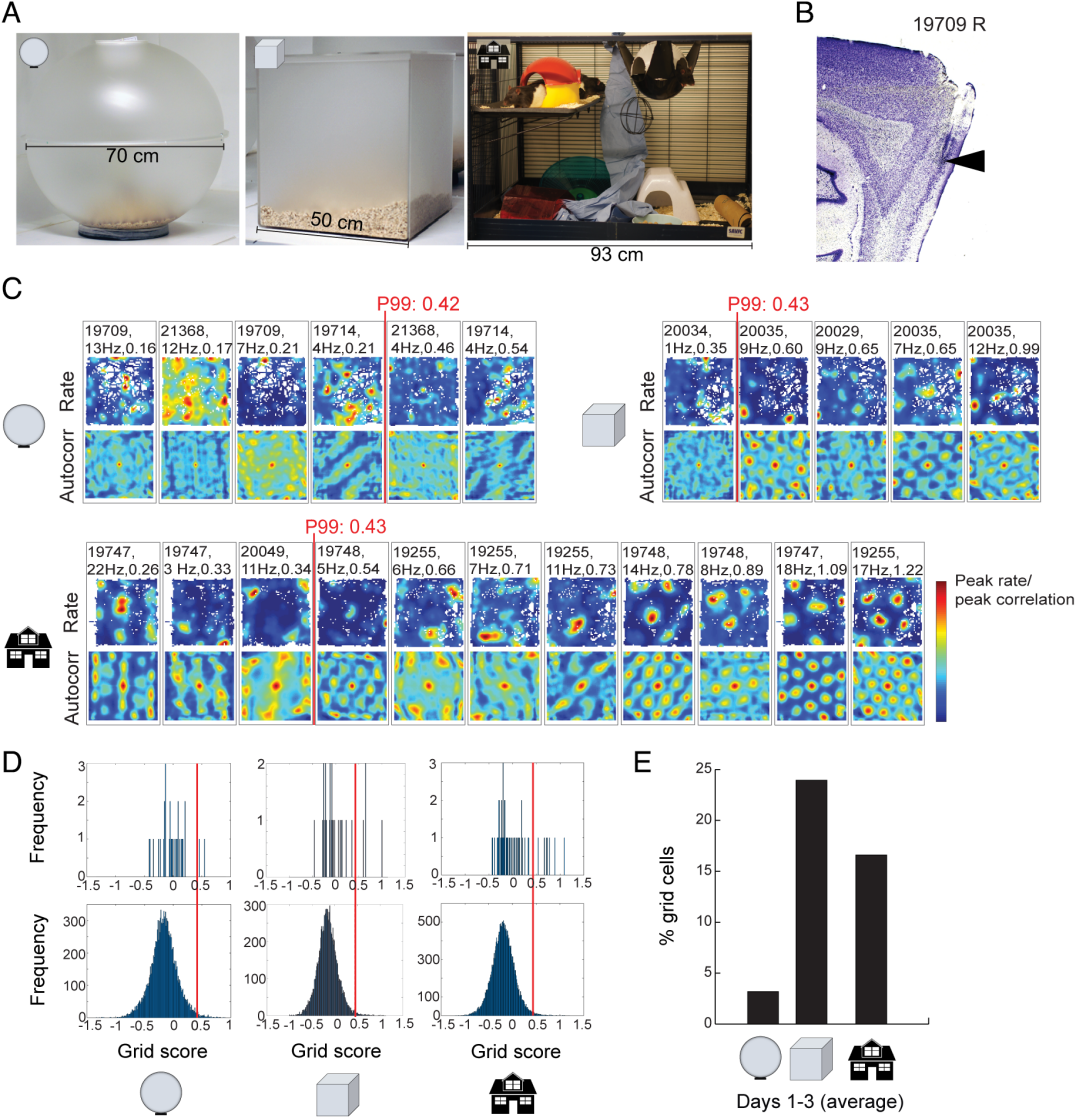
Grid cells after geometric deprivation during development. (*A*) Rats were housed in one of three types of environments: an opaque sphere (*Left*), an opaque cube (*Middle*), or a spatially enriched environment (*Right*). (*B*) Example tetrode end trace in MEC layer II of rat #19709 (R for right hemisphere). (*C*) Grid cells with spatially stable and periodic rate maps were less common in sphere-raised animals on the first day of recording outside the housing environment (150 cm arena). From left to right (low–high results): Rate maps (top row of each pair) and autocorrelation maps (bottom row of each pair) for cells that were recorded on the first encounter with the external open field (150 × 150 cm). Shown are all cells with grid scores in the upper 20th percentile of each group (top, sphere; middle, cube; bottom, spatial enrichment). Rate and autocorrelation maps are color-coded from blue (low rate, low correlation) to red (peak rate, high correlation). White areas in the rate maps show unvisited areas in the arena. Animal number, peak firing rate for the trial, and grid score are indicated for each unit at the top of the panels. Red line and text indicate the 99th-percentile criterion for grid scores based on permutation analysis. (*D*) Distribution of grid scores for rate maps of all MEC cells on Day 1 (*Top*) as well as for shuffled data (*Bottom*) from the same recordings (500 permutations per unit). Sphere group n = 31, cube group n = 27, enriched group n = 53. Red lines indicate 99th percentile of the shuffled data. (*E*) Mean percentage of cells passing the 99th percentile criterion for grid cells on Days 1 to 3 (pooled across days) in the external environment, displayed separately for the sphere group, the cube group, and the enrichment group.

Rats were born and raised for 2 to 3 mo in minimalistic cages shaped as a sphere (10 rats) or a cube (9 rats), or in a large and spatially enriched prismatic cage (5 rats) (Fig. 1*A* and *SI Appendix*, Fig. S1 *A* and *B* and Table S1). The minimalistic sphere and cube were made of opaque plastic that blocked out sharp visual contours when dim room lights were on (from 7 pm to 7 am). All husbandry or experimental preparation procedures, such as cage cleaning and neural screening recordings, took place in complete darkness (at daytime), with the experimenter wearing night vision goggles (*SI Appendix*, Fig. S1*A*).

Spike activity was recorded with tetrodes in MEC. The tetrodes were implanted after the differentially reared rats had reached an age of 6 wk or more. For the implantation surgery, animals were quickly moved, with eyes covered, to a towel-lined induction box. Moveable tetrodes were implanted in layers II-III of the dorsomedial MEC (Fig. 1*B*). After the surgery, the animals were brought to their home cages (a sphere, cube or enriched cage similar to the one they had been reared in). These home environments remained unchanged except for the removal of cage mates (for most animals; *SI Appendix*, Table S1). After 3 to 7 d of recovery in their home cages, the rats underwent cell screening and testing while resting or running in their respective home cages in complete darkness. Plugging on/off the tethered cable and recording while the rat was freely foraging was enabled by night vision goggles (*SI Appendix*, Fig. S1 *A* and *C*). The screening procedure was repeated over several days, while the tetrodes were turned in daily increments of 50 µm until distinctive theta oscillations (characteristic of MEC) were observed in the local field potential (LFP), along with high-density spike activity (characteristic of layer II of MEC).

The experiment was then started (Day 1): After an initial trial in the home cage (sphere, cube, or enriched cage), still in darkness, a second home cage trial was conducted with lights on. Next the animal was exposed for the first time to an open environment outside the rearing cage, also with lights on (*SI Appendix*, Fig. S1*C*). The open field was a large 100-cm or 150-cm-wide square box in which the rat could forage freely for cookie crumbs (100 cm box: 11 rats, 67 cells; 150 cm box: 17 rats, 158 cells). The recording was followed by a second trial in the same open field with lights on to check for stability of units. The recording day ended with a last home cage recording with lights on. This sequence of five trials (two home cage, two open field, one home cage) was repeated for up to 3 d in the cube and enrichment groups and for up to 7 d in the sphere group (*SI Appendix*, Table S1). In most animals, the tetrodes were not lowered between recordings. Analyses focused on data from the first trial of the day in the 150-cm-wide enclosure (where most of the cells were recorded and where we expected to see more grid fields per grid cell due to the size of the arena). For a limited set of data from the 100-cm box, see *SI Appendix*, Fig. S2.

Inspection of tetrode locations on Nissl-stained brain sections showed that all recordings were made in the superficial layers of the dorsomedial part of the MEC, near the border of parasubiculum, in an area known to have high densities of grid cells in rats (1, 32). In 10 rats, the tips of the tetrodes were in layer II, in 15 rats in layer III, and in 3 rats at the border between MEC layer III and the parasubiculum (*SI Appendix*, Fig. S3).

**Fig. 2.**
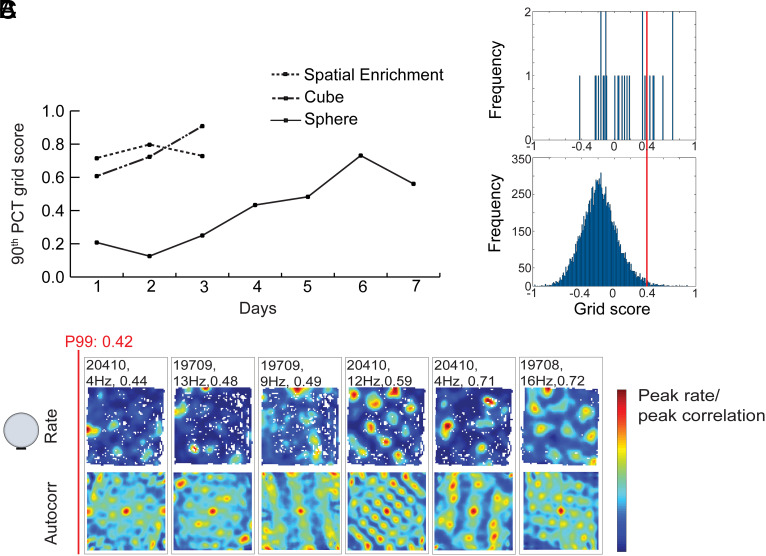
Development of grid-like firing patterns in the sphere group across 7 d of training (150 cm arena). (*A*) Line diagram showing development of 90th percentile grid scores for each group (Days 1 to 3 for cube and enrichment groups, Days 1 to 7 for the sphere group). (*B*) Distribution of grid scores for rate maps of all MEC cells on Day 7 in the sphere group (*Top*) as well as maps for shuffled data (*Bottom*) from the same recordings. Red line indicates 99th percentile of the shuffled data. (*C*) Rate maps and autocorrelation maps for cells that were recorded on Day 7 in the external open field. All cells with grid scores in the upper 20th percentile (of 28 total) are shown. The 99th percentile criterion for grid cells is shown in red. With repeated exposure to the open field, grid-like cells emerged in the sphere group.

Grid cells were identified and quantified on the basis of sixfold rotational symmetry in the cell’s firing fields (26). A cell was defined as a grid cell if the cell passed the 99th percentile value of a rotational symmetry score (grid score) for a distribution of rate maps from shuffled versions of the cells’ spike locations. During the first day of exposure to the open field, rats that had been raised in the sphere had few grid cells satisfying the criteria for spatially periodic firing fields (Fig. 1). Only 6.5% of the cells (2 out of 31) passed the criterion for grid cells during testing in the 150-cm wide box on Day 1 (grid scores of 0.46 and 0.54; criterion > 0.42; Fig. 1 *C*–*E*; note that *E* shows pooled data for Days 1 to 3). The 90th percentile grid-score of all MEC cells recorded in the 150-cm box in the sphere group was 0.21 (Fig. 2*A*). Animals from the cube and enrichment groups had a larger number of MEC cells with more clearly confined rotationally symmetric grid fields in the 150-cm box (Fig. 1 *C–E*). In total, 15.1% of the cells in the enriched group and 14.8% in the cube group passed the criterion for grid cells on Day 1 (8 out of 53 and 4 out of 27 cells) (Fig. 1 *C* and *D*). Grid scores in these groups had maximum values of 1.22 (enriched) and 0.99 (cube). For the entire MEC population in these two groups, 90th percentile scores were 0.71 and 0.61, respectively (Fig. 2*A*).

To determine if the number of spatially periodic grid cells in the sphere group increases with experience, we first monitored the animals for two additional sessions in the 150-cm open field on successive days (Fig. 2*A* and *SI Appendix*, Figs. S4 and S5). Grid-like cells were observed in the sphere group also on the second and third days but the number satisfying the spatial periodicity criterion remained low (Day 2: 0 % or 0 out of 21 cells; Day 3: 3.1 % or 1 out of 32 cells; 90th percentile grid scores for all MEC cells: 0.13 and 0.25, respectively). In contrast, in the cube group, the percentage of grid cells increased to 22.2 % (12 out of 54 cells) on Day 2 and to 12.5% (4 out of 32) on Day 3. In the enriched group, the number increased to 29.7 % (11 out of 37 cells) and 27.3% (9 out of 33), respectively. The 90th percentile grid score of the entire cell population increased on Day 2 to 0.80 and 0.72 in the cube and enriched groups, respectively, and on Day 3 to 0.73 and 0.91. Binomial tests showed that the proportion of grid cells, combined across Days 1 to 3, was significantly lower in the sphere group than in the two reference groups whereas no significant difference was observed between the cube group and the enriched group (sphere vs. enriched: Z = 2.43, *P* = 0.007; sphere vs. cube Z = 3.44, *P* < 0,001: enriched vs. cube Z = 1.40, *P* = 0.08; one-sided large-sample binomial tests; Fig. 1*C*).

The presence of weak spatial periodicity in some MEC cells of the sphere group on Day 3 led us to ask if the spatial periodicity would improve if the training was extended even further. Thus, we continued the testing for another 4 d in the sphere group. These recordings showed a gradual increase across days in the number of cells that passed the 99th percentile criterion for grid scores. Rate maps for the last day of recording—Day 7—revealed multiple cells with symmetric firing fields in the sphere group, with 6 out of 28, or 21.4%, passing the criterion (Day 7 vs. Day 1: Z = 2.02, *P* = 0.02; Fig. 2 *B* and *C*). The increase in the percentage of grid cells was accompanied by an increase in the 90th percentile of grid scores for the entire cell sample, from a value of 0.21 on Day 1 to 0.73 on Day 6 and 0.56 on Day 7, values not far off the scores recorded for the cube and enrichment groups on Day 1 (Fig. 2*A*).

**Fig. 3.**
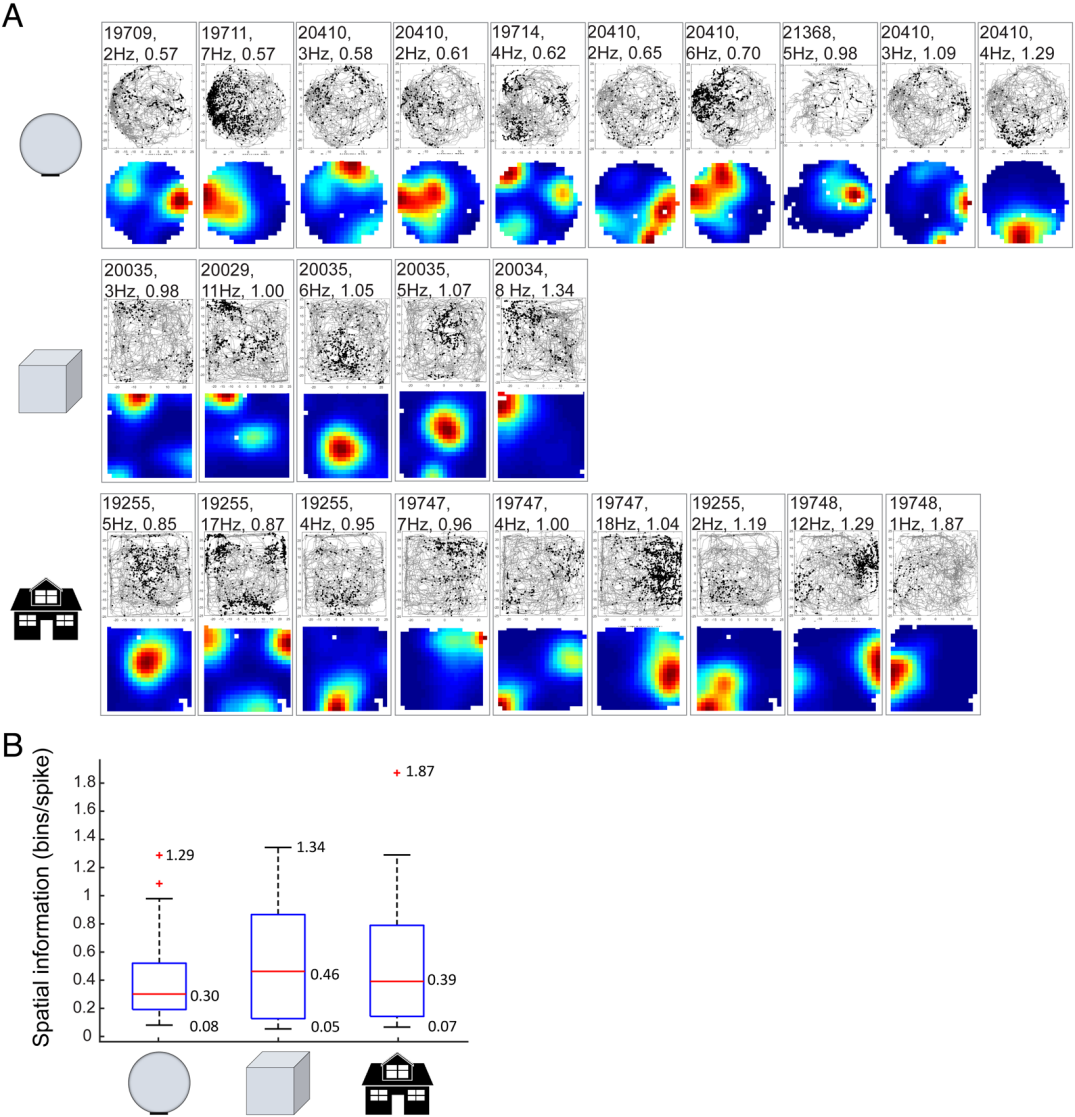
Spatially modulated firing in the home environment (home sphere, home cube, or enriched square box) before the first test in the external open field on Day 1. Data were recorded in complete darkness. (*A*) Trajectories with spike locations (*Top*) and color-coded rate maps (*Bottom*). Rows show, for each group, all cells with spatial information scores in the upper 20th percentile of the cell sample. Five-digit rat numbers, peak firing rates, and spatial information scores are indicated. Note sharp spatial tuning in all of these cells, many of which may be grid cells. (*B*) Box and whisker plots of spatial information scores (bits/spike) for each experimental group (all animals, all cells). The central line indicates the median, and the top and bottom of boxes indicate upper and lower quartiles, and the length of the whiskers indicates 1.5 times the interquartile range. Outliers are indicated by asterisks.

Since a few days of training were sufficient for grid cells in the sphere group to recover from the initial reduction in periodic firing, we asked whether the sphere-raised animals had stable firing fields within the sphere itself, where they had extensive experience. Recordings from the spherical home environment were made daily before the animal was placed in the external environment. Similar recordings were made in the cube and enrichment groups. The home environment was too small to enable detection of periodic grid fields in the environment but large enough for individual fields to be detected, should they be stable. We analyzed rate maps from the home environment on Day 1 and found multiple examples with one to three spatially confined firing fields per rate map in all three experimental groups (Fig. 3*A*). When the spatial modulation of the firing rates was estimated by a spatial information score, scores were not significantly lower in the sphere group than in the cube or enrichment group (sphere: 0.375 ± 0.035 (mean ± SEM); cube: 0.521 ± 0.080; enriched: 0.506 ± 0.061; one-way ANOVA: F(2) = 2.289, *P* = 0.106; Fig. 3*B*). The preserved spatial information scores in the spherical home environment suggests that animals can develop spatially confined firing patterns even without sharp and distinct stable environmental boundaries. It is possible, therefore, that grid structure may be expressed from early on in all animals, irrespective of experience, and that the reduction in grid structure in the new external environment occurs because it takes a few days of training for the animals to learn to anchor the grid representations consistently to an environment with a novel geometry.

**Fig. 4.**
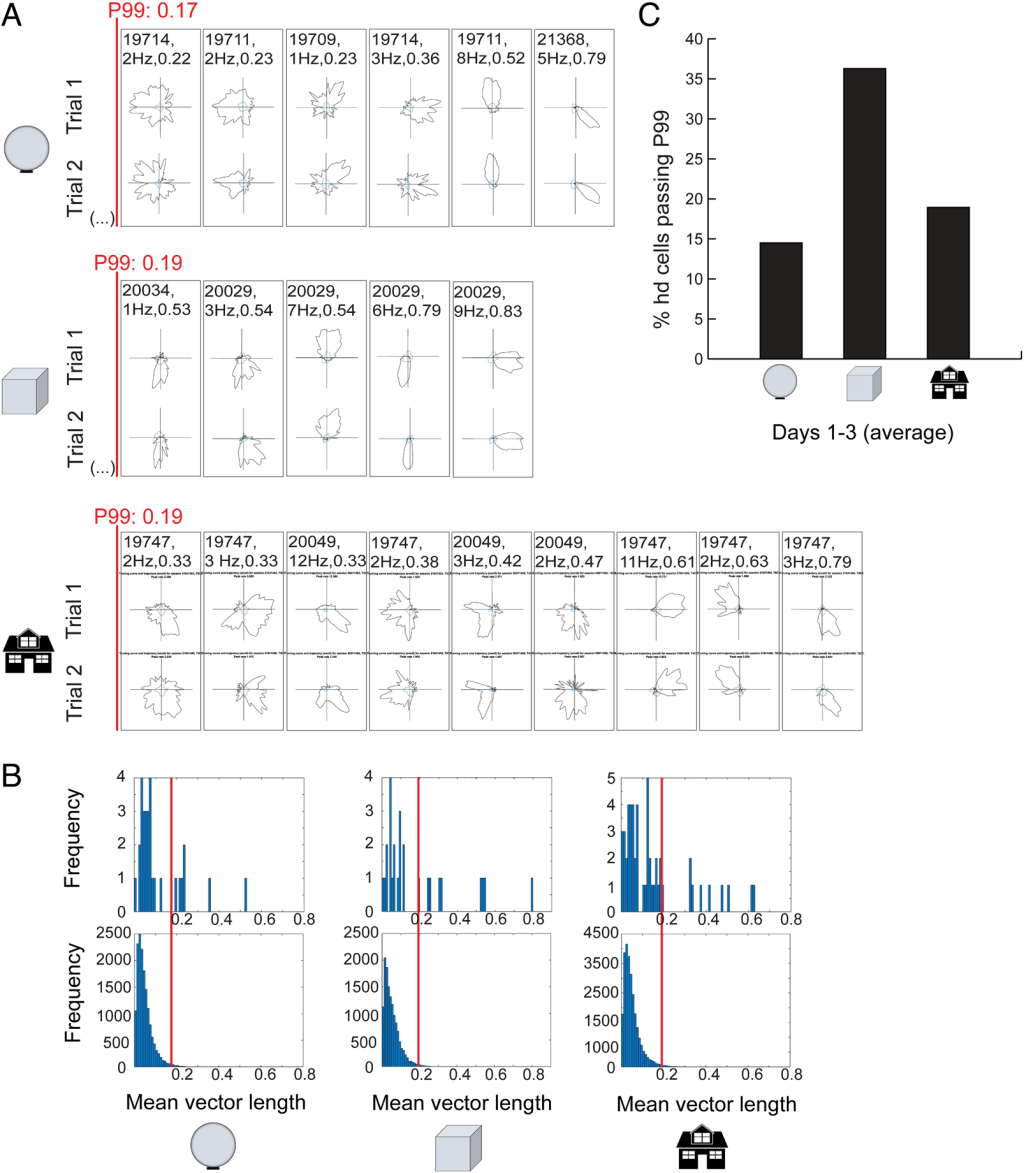
Rearing animals in the spherical environment did not impair head-direction tuning in the MEC. (*A*) Directional rate distribution for cells that were recorded in the external open field on Day 1 (gray, firing rate; blue, time spent). Top, trial 1; bottom, trial 2. The figure shows all cells with head-direction tuning in the upper 20th percentile of each group on Day 1. Directional tuning was expressed by the mvl score of the rate distribution. Top, sphere; middle, cube; bottom, spatial enrichment. Animal number, peak firing rate for the trial, and mvl score are indicated for each unit. Red line and text indicate the 99th-percentile criterion for head-direction based on permutation analysis. (*B*) Distribution of mvl for directional rate distributions of all MEC cells on Day 1 (*Top*) as well as for distributions of shuffled data from the same recordings (*Bottom*). Red lines indicate the 99th percentile of the shuffled data. (*C*) Percentage of cells passing the 99th percentile criterion for head direction for each experimental group (Days 1 to 3 pooled).

Considering that there is initial instability in the overall alignment of the entorhinal map, we asked if the impairment is present also in head direction cells, whose directional tuning patterns are normally coherent with the orientation of the grid axes (16). We tested this possibility by comparing the directional tuning of MEC cells in the three groups of the experiment on the first test in the 1.5-m-wide open field (Day 1). Directional tuning was estimated for each cell as the length of the mean vector of the cell’s directional rate distribution. There was no difference between groups in the number of head direction cells, defined as cells with mean vector lengths (mvl) exceeding the 99th percentile of a shuffled spike-position distribution (Fig. 4). A total of 22.9 % of the cells recorded on Day 1 in the sphere group (8 out of 31) passed the mean-vector-length criterion for head direction cells (Fig. 4). The maximum mvl in the sphere group was 0.79. The 90th-percentile value for mvl of all cells was 0.29. Rats that were raised in the enriched environment or in cubes had similarly tuned cells. In these animals, 20.8% and 37.0% of the cell sample passed the criterion for head direction cells (11 out of 53 and 10 out of 27 cells, respectively). The maximum mvl were 0.79 (enriched) and 0.83 (cube). For the entire cell population, 90th percentile scores were 0.43 and 0.54, respectively. The proportion of head direction cells was not significantly lower in the sphere group than in the enriched group (Z = 0.56, *P* = 0.29) or the cube group (Z = 0.92, *P* = 0.18). Enriched and cube-raised rats were also not different (Z = 1.45, *P* = 0.07). Taken together, these observations suggest that - consistent with previous observations of early maturation of head direction cells (24,25) - the orientation of the head direction map was stable from the outset in all experimental groups. Rearing in the spherical environment had more impact on phase and symmetry of grid cells than orientation of the direction map.

The delayed development of spatially periodic firing in the sphere group points to experience with geometric structure as a key factor in the rapid expression of grid patterns in new environments. To determine if such experience must occur before the MEC circuit is fully developed, we finally compared the sphere-raised juvenile animals with a group of rats that received similar housing for a similar period at adult age. Four adult animals, born and raised in a spatially enriched environment, were moved to the spherical environment at 3 mo of age and lived there for more than 10 wk. Tetrodes were implanted at the age of 5.5 mo. All handling in the sphere environment and adult sphere animals was performed in complete darkness, according to the same protocol as in the young groups.

**Fig. 5.**
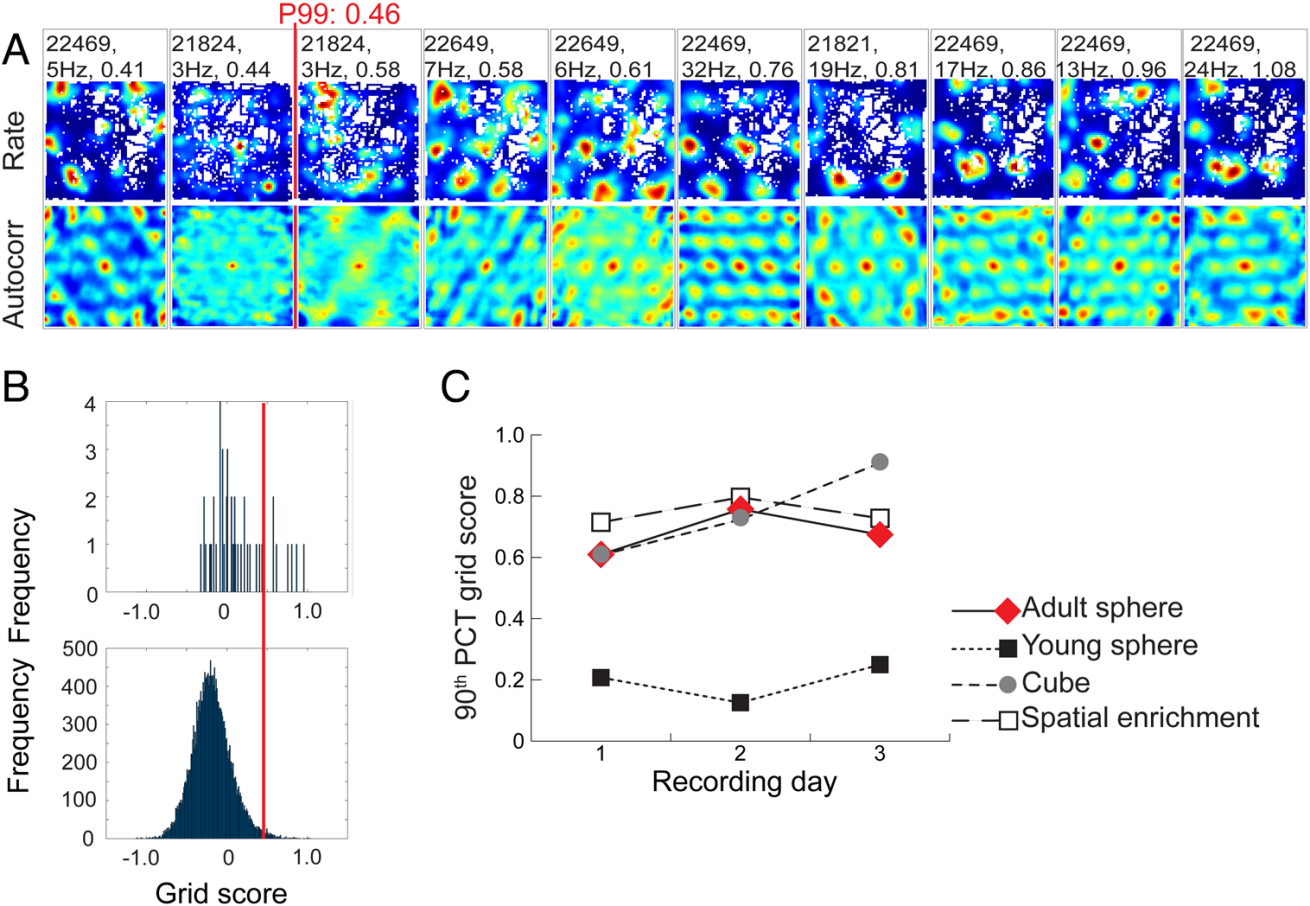
Prolonged exposure to the spherical environment did not impair grid cells in adult animals. (*A*) Rate maps (*Top*) and autocorrelation maps (*Bottom*) for cells that were recorded on Day 1 in the open field after 3 mo of continuous housing in the sphere, starting at the age of 10 wk. All cells with grid scores in the upper 20th percentile are shown. The 99th percentile criterion for grid cells is shown as a red line. (*B*) Distribution of grid scores for rate maps of all MEC cells on Day 1 in the adult sphere group (*Top*) and for maps of shuffled data from the same recording (*Bottom*). Red line indicates the 99th percentile of the shuffled data. (*C*) Line diagram showing development of 90th percentile grid scores for each group (Days 1 to 3). Data from adult rats housed in the sphere (red line) are shown along with data from the young groups (same as in Fig. 4 but here shown in dashed lines).

Confinement to the spherical environment did not cause a reduction in grid cell numbers in the adult sphere group (Fig. 5). On Day 1, 17.0% of the cells (8 out of 47) passed the criterion for grid cells (P99 = 0.46). On Days 2 and 3, the percentage of cells passing the spatial periodicity criterion was 24.5 (13 out of 53) and 12.0 (6 out of 50), respectively (P99 = 0.44 and 0.42). The 90th-percentile grid score for the entire cell sample was 0.61 on Day 1, and 0.76 and 0.67 on Days 2 and 3. The mean percentage of grid cells on Days 1 to 3 in the adult sphere group was significantly larger than in the young sphere group (Z = 3.10, *P* = 0.00097). The percentage of grid cells was not significantly different from that of the enrichment group (Z = 0.22, *P* = 0.413) or the cube group (Z = 1.32, *P* = 0.093) (Fig. 5). The fact that grid cell symmetry was not affected by deprivation of geometric reference information in the adult sphere group suggests that prior experience in spatially rich environments is sufficient for fast expression of grid cell patterns in new environments, even after months of spatial deprivation.

## Discussion

The present findings identify experience with geometric boundaries as a requirement for fast expression of spatially periodic firing patterns in MEC cells during an animal’s first encounters with a square walled environment at adult age. The formation of symmetric grid patterns in the new environment was slowed down in rats that were born and raised in an opaque plastic sphere with no vertical edges for orientation and with no access to visual contour information from the external world at any time. The impairment of grid symmetry was related specifically to the absence of stable straight vertical boundaries in the rearing environment. There was not a similar deficit in rats raised in opaque plastic cube environments that were identical to the sphere in all respects other than geometric shape. The slower emergence of symmetric grid patterns in the sphere group may reflect the absence of vertical boundaries as anchors for path integration-based navigation at young age in these animals. This impact of early experience on grid-cell representations is reminiscent of the conclusions of a long history of observations made with corresponding selective deprivation protocols in the visual system (6–15).

The dependence on straight walls for fast development of grid patterns in novel environments is consistent with observations of distorted grid patterns in adult rats foraging in environments with sharp corners and points to a prominent role for local boundaries in shaping and stabilizing the firing locations of grid cells (33, 34). The present data suggest that early experience with boundaries facilitates fast formation of crystalline grid patterns in new environments. The fact that restricted geometrical experience did not detectably impact the number of grid cells in the adult group suggests that early experience has a greater impact on map formation in a new environment than similar experience at adult age, mirroring the observations that gave rise to the concept of critical periods in the pioneering studies of visual cortex development.

At the same time as the formation of grid patterns was retarded by rearing in the sphere, it is worth noting that the animals caught up after repeated exposures to the open arena. After a week of daily training, the sphere-raised animals had approximately the same fraction of symmetric grid cells as the cube and enrichment animals had on the first exposure to the open field. Whether continued training would eventually wipe out the difference completely remains to be determined, but the data do suggest that extreme geometric rearing conditions cannot prevent the emergence of grid patterns if some experience is provided at adult age. This observation is consistent with recent neural population recordings in adult rats suggesting that the topology of the joint activity of grid cells is independent of external sensory inputs, with grid cells residing on the same toroidal manifold in sleep as during exploration, independently of ongoing experience (35). The data are also in line with observations from 3-wk-old rat pups showing that MEC cells exhibit rudimentary grid patterns, with a periodicity above the statistical chance level, after almost no explorative experience (26, 27). Fully symmetric grid patterns often do not emerge until approximately a week later in the rat’s life, but the timing of these patterns is independent of the number of training days (27), suggesting that grid cells have strong maturational components. It is thus possible that the toroidal topology of collective grid cell activity emerges independently of experience, and that experience is only required to align the torus with individual spatial environments. Not having experience with major features of environmental geometry may slow down but not prevent the alignment process. The presence of spatially tuned cells in the sphere before exposure to the external environment is consistent with this possibility since the animals were familiar with the sphere at the time of recording, although the small size of the home environments did not allow grid cells to be identified directly. The relatively limited effects of geometric deprivation in the present study are consistent with conclusions in an unpublished study of place cells showing preserved features of spatial and temporal firing profiles of hippocampal cells after rats were reared in spherical environments under similar conditions (36).

Cells samples in the present data were small since they were obtained with conventional tetrodes and microdrives but the findings were nonetheless consistent across recording days and across tests in differently sized recording enclosures (100- vs. 150-cm-wide boxes as well as recordings from within the sphere prior to exposure to the boxes). The recordings yielded too few cells to determine if the topology of the manifold of joint grid-cell activity is affected by the early experience, or if, alternatively, only the alignment with the external environment is influenced; however, with the recent development of miniaturized Neuropixels probes (35, 37), it will be possible to determine if grid cell topology is present from the earliest days of circuit maturation, before the onset of spatial exploration in the animal’s behavior.

## Materials and Methods

All animal experiments were performed in accordance with decisions laid out in the Norwegian regulation on the use of animals in experiments (FOR-2015-06-18-761) and the European Directive on the protection of animals used for scientific purposes (2010/63/EU) and in compliance with protocols approved by the Norwegian Food Safety Authorities, project license numbers FOTS 3287 and FOTS 7163.

### Subjects.

This study is based on data from 28 Long Evans rats of both sexes (10 housed in spherical environments at young age, 4 housed in spheres at adult age, 9 raised in cube-shaped environments, and 5 raised in an enriched environment). Sex and age for each individual rat is provided in *SI Appendix*, Table S1. The number of cells obtained in the respective groups was 352 (sphere, young), 332 (sphere, adult), 152 (cube), and 187 (enriched). The data were collected at the Kavli Institute for Systems Neuroscience at the Norwegian University of Science and Technology between the years 2012 and 2016.

### Housing Environments.

Pregnant breeding couples were randomly chosen to live in one of three environments, referred to as the animals’ “home cage”: i) an opaque frosted sphere with no houses or toys, ii) an opaque frosted cube with no houses or toys, or iii) an enriched multilevel wire mesh cage with houses and toys (Fig. 1*A*). Sphere and cubes were placed in a converted recording room with improved ventilation as well as temperature and humidity controls. The enriched cages were placed in regular animal holding rooms. All animals were on a reversed day/night cycle, with all lights turned off at 7 am and on at 7 pm and only dim lights turned on between 7 pm and 7 am. Light levels were made as equivalent as possible between rooms.

Pregnant breeding couples were introduced to the sphere or cube a few days prior to delivery and lived there until they were separated from the pups at postnatal day P21. Pregnant mothers were checked morning and evening to accurately assess the birthdate of litters. These checks were performed in complete darkness, with room lights turned off and the experimenter wearing night-vision goggles, to prevent visual stimulation external to the environment. One cube litter (rats #20029, 20034, 20035) was born in an enriched environment but moved with parents to the cube at P3, when the pups were blind and huddled in the nest. With large litters, litter size was reduced to a maximum of 6 to 10 pups at P3-P4 by killing the smallest pups. Litters of 6 to 10 pups lived together until implantation. In a few cases, we group-housed rats even after implantation (*SI Appendix*, Table S1).

Spheres and cubes were made of 6-mm-thick, smooth, frosted acrylic. Spheres were 100 cm or 70 cm in diameter and consisted of two equal hemispheres joined by an external lip at the equator. Cubes were 50 × 50 × 50 cm and included a perfectly fitting top lid of the same material. A circular hole of 20-cm diameter at the top of the spheres and cubes allowed for circulating air and provided a point for hanging a vertically oriented cylindrical water bottle. The water bottle was placed centrally (Fig. 1*A* and *SI Appendix*, Fig. S1*B*) to prevent its usefulness as a directional cue (unlike peripheral edges). Food pellets were randomly dispersed on the bedding floor. No chewing or housing materials or other objects were introduced in the sphere or the cube. Only for nursing litters, soft nesting paper was provided until eye opening. For some litters, a camera was set up in the top hole of the sphere and the cube to allow monitoring and recording of behavior over time. Finally, both the sphere and the cube had bedding material at the bottom, creating an absorbent floor area of similar size. While the bedding added nonuniformity to the environment, it could not, unlike the corners of the cube, be used anywhere as an unambiguous and reliable point-like reference for navigation in the rearing environment. Other rat pups of the litter or scattered food pellets could be used as references although none of these cues would be stable and reliable. Thus, while the sphere environment was not entirely stripped of navigational landmarks, the relative absence of stable and salient boundaries such as corners was sufficient to motivate a comparison of the impact of early geometrical experience on subsequent spatial map formation. The spatial enrichment cages (93 × 57 × 64 cm) were made of wire mesh and had solid floors with bedding. The cages contained several levels, running wheels, toys, hiding places, nesting-, and chewing materials.

After implantation, the rats were returned to cages as similar as possible to the ones they had been reared in, i.e., to a new sphere or a new cube (both with dimensions and content identical to the rearing sphere), or to a new enriched environment consisting of a transparent plexiglass cage (55 × 45 × 35 cm) with houses, objects, nesting and chewing materials at floor level. These postsurgery home cages were also used for screening and recording of cells in the home cage in animals of the spatial enrichment group. In 24 out of the 28 animals, cage mates were no longer present in the home cage upon return of the implanted animal (*SI Appendix*, Table S1). The remaining four animals, all belonging to the juvenile sphere group, had one cage mate in the home environment upon return. Animals in the cube and enrichment groups lived alone after surgery.

Food and water were provided ad libitum in all groups, except for mild food deprivation periods for adult animals just prior to or during experiments. The minimum amount of food provided during mild food deprivation was eight pellets (“RM1” regular maintenance, grain-based, Ø16 mm extrudate pellets, supplied by Scanbur).

All handling of rats raised or housed in a sphere or cube was performed at daytime, in complete darkness, with the experimenter wearing night vision goggles emitting infrared light. Handling and cleaning were performed exclusively by the experimenters. These measures were taken to ensure that rats in the spheres and cubes were never exposed to any visual or tactile cues outside their home cage at any time in their life, except during experiments.

Four rats were raised in the enriched environment but transferred to the spherical environment as adults, at 14 wk of age. These rats spent 10 wk in the sphere before tetrodes were implanted. They were kept in the spherical environment until the end of the experiment.

Age at the time of the experiment ranged from 6 wk to 33 wk in the young groups, with a mean age of 14.2 and 15.1 wk, respectively, in the enriched and cube groups, and 14.2 wk in the young sphere group. The adult sphere rats were 27 to 31 wk at the start of recording in the external open field.

### Surgical Procedure.

Rats were implanted unilaterally or bilaterally in the MEC with 16-channel microdrives that each were connected to four tetrodes cut flat at the same level. The tetrodes were made of 17-µm polyimide-coated platinum-iridium (90 to 10%) wire. The electrode tips were platinum-plated to reduce electrode impedances to ~200 kΩ at 1 kHz.

To induce isoflurane anesthesia, sphere and cube animals were quickly moved from the home environment to an induction box lined and covered with towels to minimize experience with corners, straight walls, or external room cues. Transfer to the induction box took <5 s. After general anesthesia was induced, the rats received analgesic drugs (buprenorphine 0.01 to 0.05 mg/kg sc for systemic analgesia and bupivacaine 1 to 3 mg/kg sc infiltrated locally at the incision site). The head was shaved, and the skin was cleaned with 70% ethanol and iodine. Throughout the surgery, we hydrated the surgery subjects with subcutaneous injections of sterile saline, and an electrical heat pad ensured a core body temperature of 37 °C. The eyes were covered with Simplex Vaseline ointment to prevent damage. After induction of anesthesia, the rats were moved to a stereotaxic apparatus (Kopf) where the head was immobilized with blunt ear bars and a mask. A medial incision was made in the skin from the rostral to the caudal part of the skull, exposing the skull’s landmark sutures, bregma, and lambda. The skull bone was exposed and cleaned with sterile saline, and the skin was retracted with hemostats. Skull landmarks were aligned to the azimuth to ensure repeatability of implantations. To secure proper stability of the implant, we used a dental drill to make burr holes and anchored jewellers screws in the skull bone or applied dental Optibond. A craniotomy was made 4.5 to 4.7 mm from the midline in the mediolateral plane just caudal to the posterior transverse suture, exposing the transverse sinus. The dura mater anterior to and over the sinus was punctured or removed, and the tetrodes were lowered toward the dorsomedial entorhinal cortex, at 0.1 to 0.3 mm anterior to the sinus and 1.8 to 2.0 mm in the dorsoventral plane, with a 10 to 20 degrees forward angle of the drive. The tetrodes were protected by a 19-gauge cannula and sterile Spongostan Dental (Ethicon) that surrounded the cannula and covered the remaining space in the craniotomy. The microdrive was cemented in place and the skull surface was covered with dental cement. The skin was released to close the wound to the smooth-edged cement. In four animals that were cohoused with a cage mate after surgery, cement and tape, or a plastic removable casing, were added to protect the implant during social interactions in the home cage. After the implantation the rat was taken off the anesthesia but kept warm and hydrated until the initial reflexes were gained. The animal was then moved to its home cage, and provided with soft foods, water, and medication as needed in the recovery period. For postsurgical analgesia, meloxicam/Metacam was administered orally (1.5 mg/mL), at the dosage recommended for 24 h pain relief (1 mg/kg body weight).

### Cell Screening and Recording of Neural Activity.

After 3 to 7 d of postsurgical recovery, implanted rats were plugged in for cell screening while foraging in their home cage. In all groups, plugging cables on or off, cell screening, and turning of tetrodes were performed in complete darkness with the experimenter wearing night vision goggles. During screening, to ensure that the animals were not unintentionally exposed to light from outside sources, e.g., through the crack of a door, the home cage was placed in a section of the recording room without doors or windows. The room was then divided into a dark and a dim light condition by setting up a room-divider made up of multiple layers of light-proof curtains from floor to ceiling. Cell screening was conducted in the dark for a minimum of 1 d to a maximum of 18 d, with a mean of 8 d. A single or double cable connected the animal’s microdrive to an AC-coupled unity-gain operational amplifier during these procedures. The weight of the cable was counterbalanced via a pulley system. There was no food deprivation during trials in the home cage.

Recorded signals were amplified 4,000 to 12,000 times and band-pass filtered between 0.8 and 6.7 kHz. Triggered spikes were stored to disk at 48 kHz with a 32 bits time stamp. An overhead camera recorded the position of one large and one small light-emitting-diode (LED) on the head stage. The diodes were positioned 5 cm apart and aligned with the body axis. After each session of cell screening, cluster-cutting and cluster analysis was carried out. When theta oscillations and stable clusters were present in the screening trial, the experiment in the external environment was ready to be started.

### Recording Outside the Housing Environment.

When stable cells had been obtained in the home cage screening recordings, and tetrodes were judged to be within MEC (based on theta oscillations and dense activity characteristic of MEC layer II), we started recordings in a novel arena outside the housing environment. From this point on, the animals were mildly food-deprived at the time of testing (they received eight pellets per 24 h; pellets were made available 24 h before testing). Each experimental session consisted of five trials: first two 10-min trials in the home cage (the first in complete darkness, the second with soft room lights on), then two 20 to 45-min trials in an open-field environment with lights on, and finally another 10- to 15-min trial in the home cage with lights on. The interval between trials was spent in the home cage and lasted 15 to 30 min. The two initial trials in the home cage ensured that recorded units were stable with respect to waveform shapes and firing properties. On the third and fourth trials, the rats were exposed, for the first time, to a black square open-field enclosure, which was either 150 × 150 × 50 cm or 100 × 100 × 50 cm.^.^ All but two rats were tested only in one box size (150 cm arena: four spatially enriched rats, four cube rats, and five sphere rats during early development, plus four rats that experienced the sphere at adult age; 100 cm arena: one spatially enriched rat, five cube rats, and three sphere rats during early development). For two young sphere rats initially recorded in the novel 1-m box for two or four sessions (rats 18655 and 29410), we changed to a novel 1.5-m box (same recording room/location) on Days 3 and 5, respectively (*SI Appendix*, Table S1). This move was needed to record more grid fields than was visible at first in the smaller box. During the experiments, the rats were kept engaged in free foraging behavior by throwing cookie crumbles randomly into the open field. Stable cues were visible to the rat; one A3-sized white cue card on the curtains was visible from the home cage, while an additional white A4-sized cue card was visible on the north wall of the open field. The locations of the open field and the cue cards, and their relative position to the home cage, were constant. The rats were always released from the same corner in the box. The box was cleaned with soapy water between each trial to remove odor cues. Between trials, the rats rested next to the open arena in their home environments with lights off. The first encounter the rats had with the novel square arena was called “Day 1,” the next encounter “Day 2,” etc. Cube and spatial enrichment rats were tested for 3 d, while sphere-raised rats were tested up to 7 d.

### Spike Clustering and Classification.

Cell classification was performed manually using graphical cluster cutting tools as described previously (26). Clusters recorded on different days were overlapping, implying that the same cells were often recorded in cell samples from different days. The rat’s position was tracked via LEDs on the rat’s headstage. All data were speed filtered (epochs with speed lower than 2.5 cm/s or higher than 100 cm/s were deleted). Position data were smoothed using a 21-sample boxcar window filter (400 ms, 10 samples on each side).

### Rate Maps.

Firing rate distributions were determined by counting the number of spikes and time spent in each 2.5 cm × 2.5 cm bin, using a boxcar average over the surrounding 5 × 5 bins (26). To improve the trade-off between blurring error and sampling error, an adaptive smoothing method was used on the rate maps before field size and grid scores were estimated (26, 38).

### Analysis of Grid Cells.

The periodicity of the rate maps was evaluated for all cells with average rates above 0.20 Hz by calculating a spatial autocorrelation map for each smoothed rate map (5). The degree of spatial periodicity was determined for each recorded cell by taking a central circular sample of the autocorrelogram, with the central peak excluded, and comparing rotated versions of this sample (5, 26). The Pearson correlation of the circular sample with its rotation in α degrees was obtained for angles of 60° and 120° on one side and 30°, 90°, and 150° on the other. The cell’s grid score was defined as the minimum difference between any of the elements in the first group and any of the elements in the second. Grid cells were identified as cells in which rotational-symmetry-based grid scores exceeded the 99th percentiles of distributions of grid scores, respectively, in shuffled versions of the same data, with all cells from each group shuffled together following procedures described previously (26). Shuffling was performed with 500 permutation trials per recorded cell. Grid scores were computed for all permutations for all cells, and the 99th percentile was extracted from the joint distribution. Cells with grid scores above this threshold in the data were defined as grid cells.

### Analysis of Head Direction Cells.

The directional tuning function for each cell was obtained by plotting the firing rate as a function of the rat’s directional heading, divided into bins of 0.5 degrees. For each directional bin, the number of spikes and the time spent in the bin was determined, after smoothing the maps for number of spikes and time individually with a 14.5 degrees mean window filter (14 bins on each side). The degree of directional tuning was estimated by computing the length of the mean vector for the circular distribution of firing rate (number of spikes divided by time). Head direction-modulated cells were defined as cells with mvl exceeding the degree of directional tuning that would be expected by chance. The chance level was determined for each experimental group by a time-shift shuffling procedure equivalent to the one used for grid cells. Permutations were performed for individual cells, 500 times for each cell. For each permutation, a head direction tuning curve was constructed, and the mvl was calculated. The distribution of mvl was computed for the entire set of permutations from all cells in the sample/group, and the 99th percentile was determined and used as a cutoff for head direction cells.

### Histology and Immunohistochemistry.

The tetrodes were not moved after the last recording day. The rat was deeply anesthetized, received an overdose of Pentobarbital, and was perfused with an intracardial injection of 9% saline followed by 4% formaldehyde. Tetrodes were left in the brain for 30 to 60 min after perfusion. After removing the brain from the skull, the brain was stored in 4% formaldehyde and postfixed for several days. The brain was then quickly frozen and cut into 30-mm sagittal slices, mounted on glass, and stained with cresyl violet. The final position of the tip of each tetrode was identified in the microscope or on digital pictures of the brain sections. All rats reported in this study had tetrode tips in the superficial layers of the MEC or MEC/parasubicular border.

## Supplementary Material

Appendix 01 (PDF)

## Data Availability

Spike-time data have been deposited in EBRAINS (https://doi.org/10.25493/KXEN-EAF) (39).
