# Supplementary material for: Grid cells in rats deprived of geometric experience during development
Source: Proc Natl Acad Sci U S A. 2023 Oct 2;120(41):e2310820120. doi: 10.1073/pnas.2310820120 (PMC10576132; doi:10.1073/pnas.2310820120)
Supplement: Supplementary file 1 — Appendix 01 (PDF) [file pnas.2310820120.sapp.pdf]

PNAS

Supporting Information for

Grid cells in rats deprived of geometric experience during development

Ingvild Ulsaker-Janke, Torgeir Waaga, Tanja Waaga, Edvard I. Moser, May-Britt Moser

Corresponding authors: Ingvild Ulsaker-Janke, ingvild.ulsaker-janke@ntnu.no; May-Britt Moser, maybm@ntnu.no

This PDF file includes:

Figures S1 to S5

Table S1

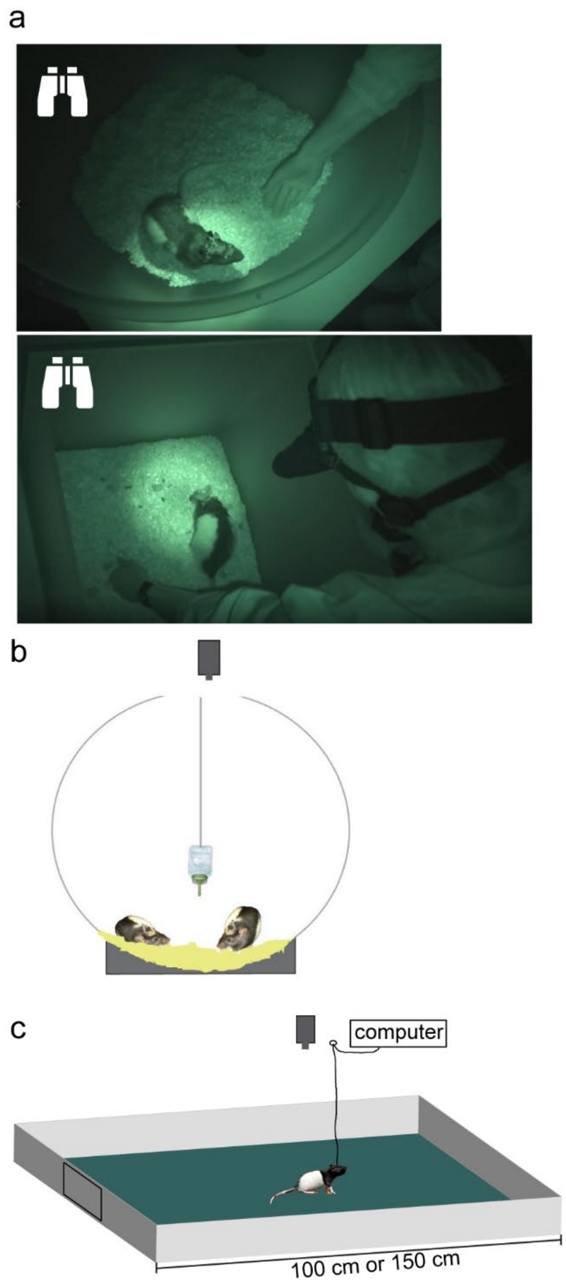

Figure S1. Experimental protocols. **a**. Activity was recorded daily while the rats foraged in their home cage in complete darkness. The procedure was maintained until the tetrodes had reached layers III-II of the MEC. Home sphere (top) and home cube (bottom) in darkness shown here, prior to plugging on the recording cable. Home enriched box is not included here. See materials and methods paragraph on housing environments for details. **b**. Cross-section of spherical environment showing central position of water bottle. **c**. When theta oscillations and dense spike activity were present at the estimated location in MEC, the experiment started and the rat was released for the first time in an environment – a square open field – external to the home environment.

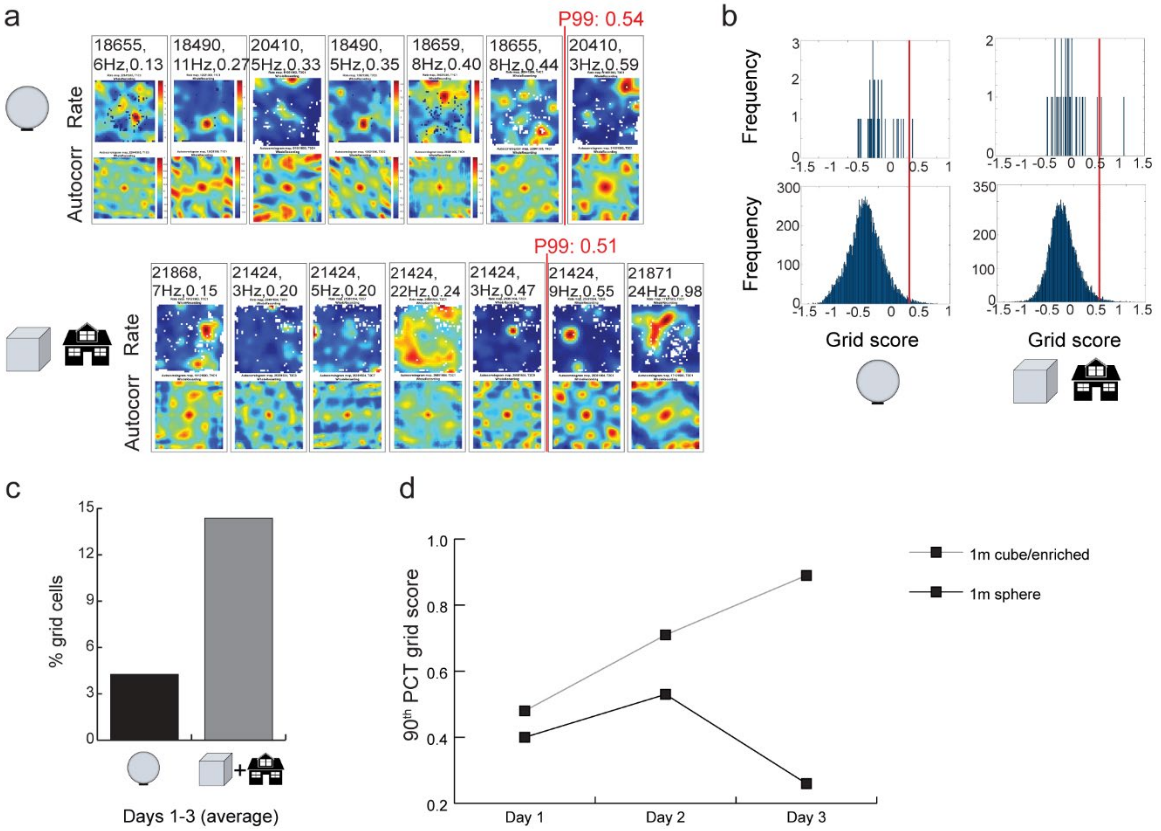

Figure S2. Effects of rearing environment in animals tested in the smaller box (100 cm width). **a.** From left to right (low – high results): Rate maps (top row of each pair) and autocorrelation maps (bottom row of each pair) for cells that were recorded on the first encounter with the external open field (100 × 100 cm). Shown are all cells with grid scores in the upper 20th percentile of each group (top, sphere; bottom, cube and spatial enrichment combined). Rate and autocorrelation maps are colour-coded from blue (low rate, low correlation) to red (peak rate, high correlation). Animal number, peak firing rate for the trial, and grid score are indicated for each unit. Red line and text indicate 99th-percentile criterion for grid scores based on permutation analysis. See also Supplementary Table for overview of animals and cells. **b**. Distribution of grid scores for rate maps of all MEC cells on Day 1 (top) as well as maps for shuffled data from the same recordings (500 permutations per unit). Sphere group n = 33, cube group and enriched group n = 34. Red lines indicate 99th percentile of the shuffled data. **c**. Mean percentage of cells passing the 99th percentile criterion for grid cells on Days 1-3 (pooled) in the external environment, displayed separately for the sphere group and combined cube- and enrichment group. **d.** Line diagram showing development of 90th percentile grid scores for sphere group versus combined cube and enrichment group (Days 1-3).

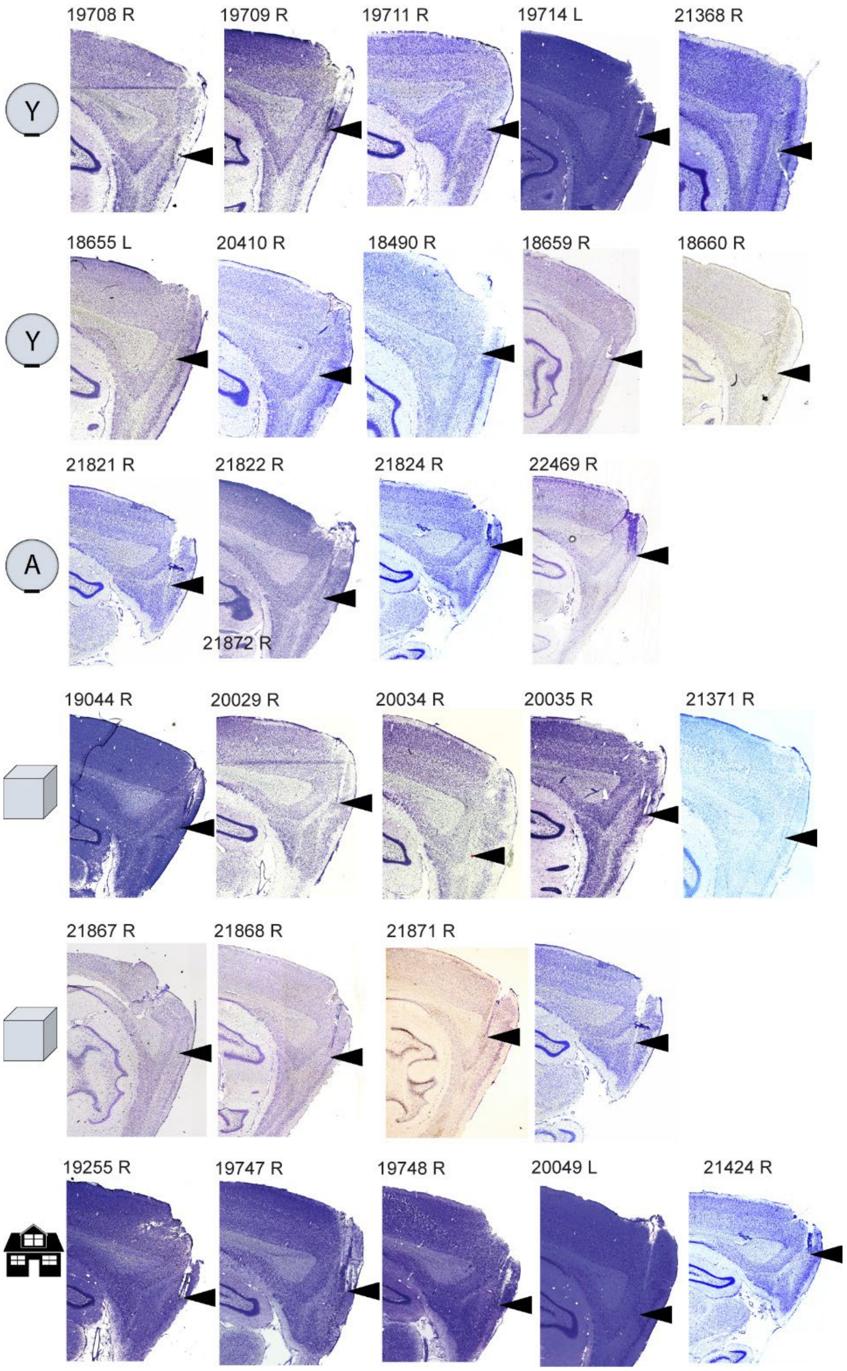

Figure S3. Recording locations for all animals recorded in 1 m^2 or 1.5 m^2 open field. One Nissl-stained sagittal section is shown for each individual rat (the section with the deepest tetrode trace). From top: young sphere group (n = 10); adult sphere group (n = 4); cube group (n= 9); spatial enrichment group (n = 5). Rat numbers are indicated (5 digits). R and L, right and left hemispheres, respectively. Black arrowheads indicate the recording location at the bottom of the tetrode trace. Tetrodes were not moved after data collection ended.

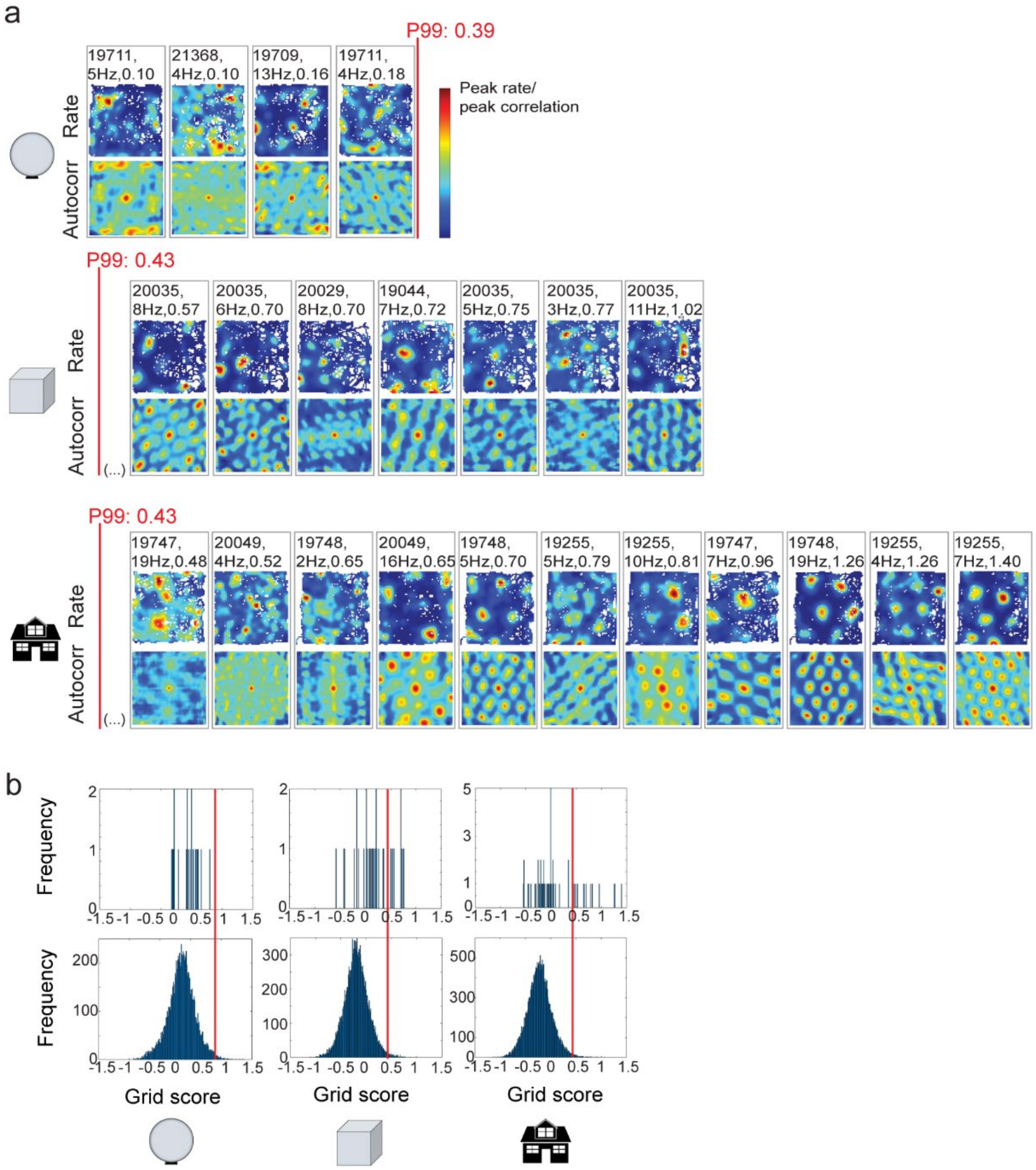

Figure S4. Grid-like firing patterns on Day 2 in the external open field (150 cm box). **a**. Rate maps and autocorrelation maps. All cells with grid scores in the upper 20th percentile are displayed. 99th percentile criterion for grid cells is shown in red. In the sphere group no cells passed the criterion for grid cells. **b**. Distribution of grid scores for rate maps of all MEC cells on Day 2 (top) and for maps of shuffled data from the same recordings. Red line indicates 99th percentile of the shuffled data.

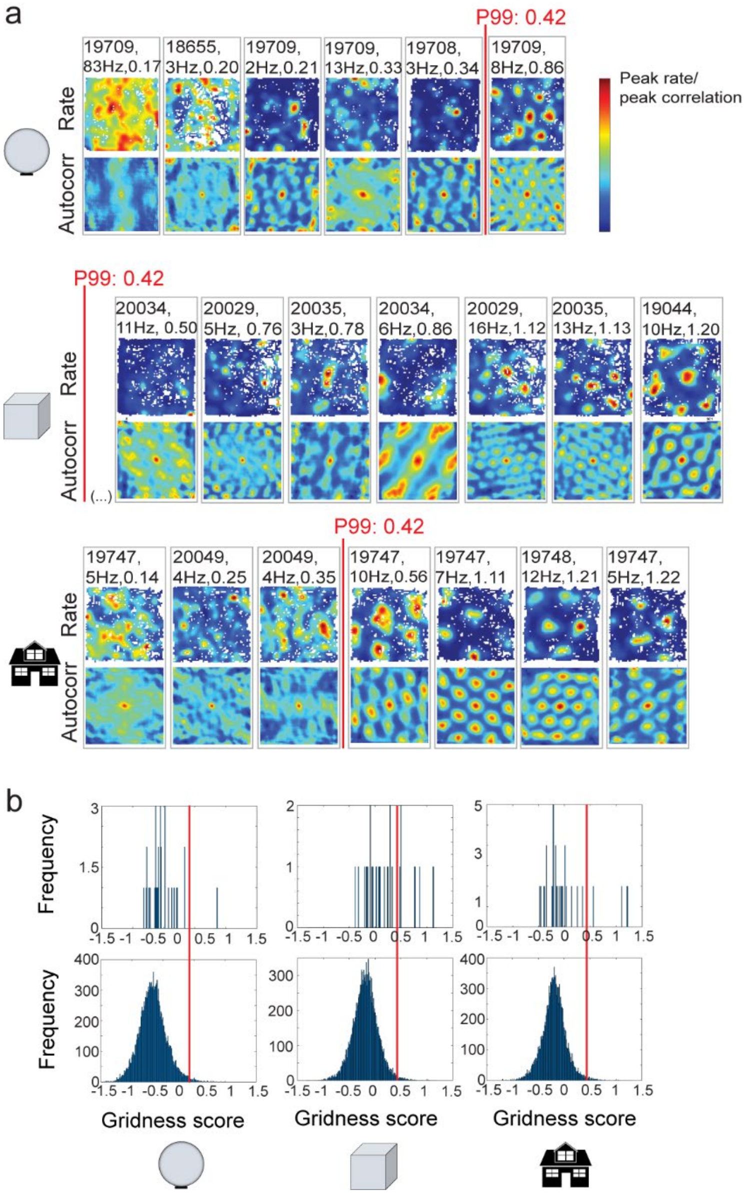

Figure S5. Grid-like firing patterns on Day 3 in the external open field (150 cm box). **a**. Rate maps and autocorrelation maps. All cells with grid scores in the upper 20th percentile are displayed. 99th percentile criterion for grid cells is shown in red. In the sphere group one cell passed the criterion for grid cells. **b**. Distribution of grid scores for rate maps of all MEC cells on Day 3 (top) and for maps of shuffled data from the same recordings. Red line indicates 99th percentile of the shuffled data.

Rats per group	Group	Rat #	Sex	Rec. age (weeks)	Units							BOX SIZE	
					day 1	day 2	day 3	day 4	day 5	day 6	day 7	1 m	1.5 m
		19708	F	11	1	2	3	1	1	1	*		x
		19709	F	8	12	11	12	13	10	12	13		x
		19711	M	15	5	3	*	*	*	*	*		x
		19714	M	21	8	3	3	4	*	*	*		x
10	Young sphere	21368	M	16	5	5	3	*	*	*	*		x
		18655†	M	14	12	12	11	8	9	9	*	x	x
		20410	M	33	8	9	7	14	12	12	13	x	x
		18490†	F	9	4	6	5	7	*	*	*	x	
		18659†	F	7	6	10	11	*	*	*	*	x	
		18660†	F	8	3	4	7	9	8	5	*	x	
		Sum of units			64	65	62	56	40	39	26		
		21821	M	27	11	10	12	11	10	10	*		x
4	Adult sphere	21822	M	31	3	10	11	13	12	13	11		x
		21824	M	27	16	19	15	11	13	12	14		x
		22469	M	26	17	14	12	13	13	13	13		x
		Sum of units			47	53	50	48	48	48	38		
		19044	M	21	1	12	7						x
		20029	F	12	10	11	12						x
		20034	M	14	5	5	5						x
		20035	M	20	11	9	10						x
9	Cube	21371	M	6	6	10	*					x	
		21867	M	19	1	**	1					x	
		21868	M	14	6	6	5					x	
		21871	F	15	3	2	*					x	
		21872	F	15	5	5	4					x	
		Sum of units			48	60	44						
		19255	M	18	10	11	*						x
		19747	M	14	18	16	7						x
5	Enriched	19748	M	12	11	14	11						x
		20049	F	10	14	14	14						x
		21424`	M	17	13	17	17					x	
		Sum of units			66	72	49						

Discrepancies in the sum of cells given here and in results section are due to incalculable score values (NaNs: 3 cells on Day 2 in the young sphere group; 1 cell on Day 3 in the cube group; 1 cell on Day 2 in the enriched group).

* = recording discontinued
** = poor coverage
21424` = on Day 1 jumping and poor coverage in 1st box trial, so 2nd box trial from Day 1 is included here
† = housed with cage mate also after implantation.

Table S1. Experimental protocol for each animal of each experimental group with differential juvenile or adult housing (number of animals in each group, group, rat number, rat name, number of cells recorded per day, and size of novel open field arena).
